# Advanced Nanomaterials for Quantum Technology, Sensor and Health Therapy Applications

**DOI:** 10.3390/nano13091506

**Published:** 2023-04-28

**Authors:** Sotirios Baskoutas

**Affiliations:** Department of Materials Science, University of Patras, 26500 Patras, Greece; bask@upatras.gr

The intense interest in nanostructured materials is fueled by the tremendous economic and technological benefits anticipated to be achieved by nanotechnology and nanodevices. Nanostructured materials have demonstrated great potential for applications in optoelectronics, sensors and cancer therapy. Advances in these areas will affect our daily life, ranging from how we design a fast computer to how we preserve the environment, and how we diagnose and treat disease and pollution.

This Special Issue aims to cover a broad range of subjects, ranging from nanomaterials for quantum technology applications to sensor, solar cells and health science applications.

In this Special Issue, there are research articles that focus on the use of nanomaterials for quantum technology applications [1,2,3,4,5,6,7], nanomaterials for health science [8,9,10] and nanomaterials for solar cells [11].

Finally, I would like to express my sincere gratitude to all the authors who contributed their innovative research to this Special Issue.
